# Comparison of combinations of irradiation techniques and jaw conditions in intensity‐modulated radiotherapy for lung cancer

**DOI:** 10.1002/acm2.13416

**Published:** 2021-09-10

**Authors:** Qinghe Peng, Junyue Shi, Jun Zhang, Qiwen Li, Zhenghuan Li, Qingyuan Zhang, Yinglin Peng, Li Chen

**Affiliations:** ^1^ Department of Radiation Oncology Sun Yat‐sen University Cancer Center, State Key Laboratory of Oncology in South China, Collaborative Innovation Center for Cancer Medicine Guangzhou China; ^2^ Department of Radiation Oncology Foresea Life Insurance Guangzhou General Hospital Guangzhou China; ^3^ Department of Radiation Oncology the Third Affiliated Hospital, Sun Yat‐sen University Guangzhou China; ^4^ Department of Radiation Oncology Guangzhou Concord Cancer Center Guangzhou China

**Keywords:** adaptive jaw, IMRT, jaw tracking, lung cancer, VMAT

## Abstract

**Purpose:**

To assist in the selection of a suitable combination of an irradiation technique and jaw condition in intensity‐modulated radiotherapy (IMRT) and volumetric‐modulated arc radiotherapy (VMAT) for lung cancer treatment plans.

**Materials and methods:**

Thirty patients with lung cancer who underwent radiotherapy were enrolled retrospectively. They were categorized as having central lung cancer, peripheral lung cancer with mediastinal lymph node metastasis (peripheral E lung cancer), and peripheral lung cancer without mediastinal lymph node metastasis (peripheral N lung cancer). Four treatment plans were designed for each patient: fixed jaw and adaptive jaw IMRT technique (FJ‐IMRT and JA‐IMRT), and fixed jaw and jaw tracking VMAT technique (FJ‐VMAT and JT‐VMAT). The dose parameters of the four group plans were compared and analyzed.

**Results:**

Compared to FJ‐IMRT, JA‐IMRT significantly reduced the mean dose (D_mean_) and volume percentage of 5 Gy (V_5Gy_) of the total lung in central and peripheral N lung cancer. Similarly, compared to FJ‐VMAT, JT‐VMAT provided better protection to most organs at risk (OARs), particularly for total lung and heart. In comparison with IMRT, VMAT significantly improved the conformity index (CI) of the planning target volume for the three lung cancer classifications, and it reduced the dose of almost all OARs except V_5Gy_ and D_mean_ of the total lung. Moreover, the mean monitor units of the VMAT groups were far lower than the IMRT groups.

**Conclusion:**

Based on the dosimetric findings and considering clinical data published on lung and heart side effects, we propose recommendations on the preferred treatment technique based on tumor location and pulmonary function. For central lung cancer with normal pulmonary function, we advise JT‐VMAT techniques. Conversely, for central lung cancer with poor pulmonary function, we recommend JA‐IMRT techniques. We advocate JA‐IMRT for peripheral E lung cancer. For peripheral N lung cancer, JT‐VMAT techniques are strongly recommended.

## 1 INTRODUCTION

For lung cancer, intensity‐modulated radiotherapy (IMRT) and volumetric‐modulated arc radiotherapy (VMAT) techniques have a significant dosimetric advantage over 3D conformal radiation therapy.^1^, ^2^, ^3^, ^4^, ^5^ Several studies have focused on dosimetric differences between IMRT and VMAT plans for lung cancer.^6^, ^7^, ^8^ Li et al.^9^ discovered specific advantages of various IMRT and VMAT strategies for lung cancer in different locations and proposed the application of different planning techniques for lung cancer treatment according to the classification—central or peripheral—and the requirements of the organs at risk (OARs). Wang et al.^10^ indicated that in the application of the IMRT technique, the adaptive jaw (JA) was superior to the fixed jaw (FJ) plan in decreasing the radiation exposure of surrounding OARs. In Varian linear accelerators, such as VitalBeam (Varian Medical Systems, Palo Alto, CA, USA), a jaw tracking (JT) mode can be applied for VMAT. The JT mode can minimize leakage and transmission through the multileaf collimator (MLC) leaves by the jaws following the MLC apertures.^11^ Further research has confirmed the advantages of the JT mode for significantly reducing doses to the OARs and normal tissues around the target.^12^, ^13^, ^14^, ^15^, ^16^ However, few studies have surveyed the dosimetric impact of different jaw conditions combined with different irradiation techniques for lung cancer in various locations. Therefore, in the study, we compared the dosimetric differences of the four radiotherapy techniques for the three lung cancer categories in order to assist in the selection of a suitable combination of an irradiation technique and jaw condition in (IMRT and VMAT for lung cancer treatment plans.

## 2 MATERIALS AND METHODS

### 2.1 Patients’ clinical characteristics

A total of 30 patients with lung cancer—10 with central lung cancer, 10 with peripheral lung cancer with mediastinal lymph node metastasis (peripheral E lung cancer), and 10 with peripheral lung cancer without mediastinal lymph node metastasis (peripheral N lung cancer)—who underwent radiotherapy from May 2020 to September 2020 at the Sun Yat‐sen University Cancer Center (Guangzhou, China) were randomly selected. All patients were staged in accordance with the modified 1997 American Joint Committee on Cancer staging system. The patient characteristics are listed in Table 1.

**TABLE 1:** Patient characteristics (*n* = 30)

**Characteristic**	**Statistic**
**Age (years)**	
Median	58
Range	31–72
**Sex (no. of patients)**	
Male	25
Female	5
**Disease stage**	
II	10
IIIa	3
IIIb	10
IIIc	7
**Cancer category**	
Central (198.9–681.9 cm^3^)	10
Peripheral E (206.8–918.2 cm^3^)	10
Peripheral N (46.1–150.8 cm^3^)	10

*Notes*: Peripheral E refers to peripheral cases with mediastinal lymph node metastasis; Peripheral N refers to peripheral cases without mediastinal lymph node metastasis. The volume of the planning target volume (PTV) for the category of lung cancer is indicated in parentheses.

### 2.2 Image acquisition

All patients, fixed with a vacuum bag, were placed in a supine position, headfirst. Then, 4D contrast‐enhanced helical computed tomography (CT) scans were then performed by a CT Simulator system (Philips; Brilliance BigBore) and respiratory gating system (RPM, Version 1.7; Varian) The RPM system monitored the patient's breathing cycle and obtained 10 sets of CT images of different breathing phases according to the breathing cycle curve scan. The CT images acquired in this manner were transmitted to the treatment planning system (Monaco 5.1; Elekta AB, Stockholm, Sweden) to delineate the target volumes and OARs.

### 2.3 Delineations of target volumes and OARs

According to the location of the primary tumor and lymph node metastasis, the cases are divided into three categories: central type, peripheral E type, and peripheral N type. Central type means that the tumor is located in the center of the lung and occurs above the tertiary bronchus. Peripheral lung cancer means the tumor is located in the periphery of the lung and occurs below the tertiary bronchus, and those with mediastinal lymph node metastasis are peripheral E type, otherwise, they are peripheral N type. The target volumes for all patients were delineated by an experienced radiation oncologist according to the Radiation Therapy Oncology Group guidelines.^17^ The gross tumor volume (GTV) was defined by the visualization of all gross tumors and the involved lymph nodes. The clinic target volume (CTV) was defined as the potential volume harboring microscopic disease. Following the delineation of the GTV and CTV, the corresponding planning target volume (PTV), respectively referred to as the PGTV and PCTV, were generated by using margin expansion to account for positioning errors. The delineated OARs include the total lung, spinal cord, esophagus, and heart.

### 2.4 Treatment planning

**FIGURE 1:**
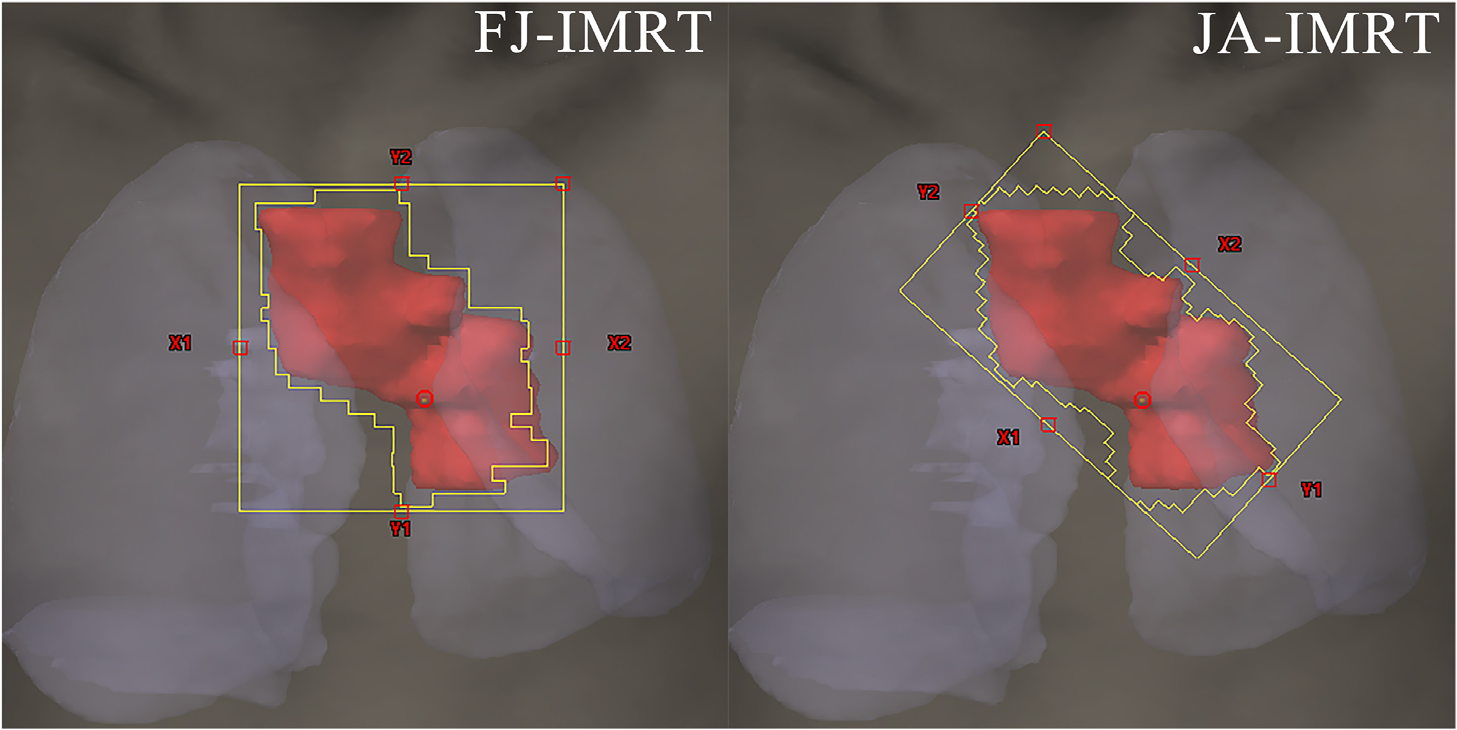
Jaw condition of the intensity‐modulated radiotherapy (IMRT) plans: (a) Fixed jaw IMRT plans and (b) adaptive jaw IMRT plans

The CT images and contoured structures of each patient were transmitted to the treatment planning system (Eclipse 15.5; Varian) for the plan design. Four plans were designed for each patient using the FJ and JA combined with the dynamic MLC (d‐MLC) IMRT technique (the FJ‐IMRT and JA‐IMRT plans, respectively) and then the FJ and JT modes combined with the d‐MLC VMAT technique (the FJ‐VMAT and JT‐VMAT plans, respectively). The prescribed doses to the PGTV and PCTV were respectively 60 and 50 Gy in 30 fractions. The optimization objectives and constraints described in Table 2 were the same for the four techniques. The FJ‐IMRT plans were created using sliding window dynamic delivery and five fixed beam angles, and the collimator angle of all five beams was 0° and the length and width of the jaw were automatically adjusted by the treatment planning system. (Figure 1a). The collimator angle and jaw positions of the JA‐IMRT plans were manually optimized according to the target shape to keep the minimum size of the jaw in the case of ensuring the coverage of the target area (Figure 1b). The FJ‐VMAT plans were using VMAT with FJ width and length, which were designed with two complementary coplanar arcs of 360° (one counterclockwise from 179° to 181° and the other clockwise from 181° to 179°) for the central cases and peripheral E cases, and two partial arcs for the peripheral N cases. The collimator angles of the two arcs were 15° and 345°. The JT‐VMAT plans were designed with the jaw technique switched to the automatic tracking mode. All plans were selected with 6 MV energy photons and the machine and optimization parameters were identical. Moreover, an anisotropic analytical algorithm (version 15.5.12) with a dose calculation grid of 2.5 mm was used to calculate the volumetric doses. The reference volume for the treatment planning was the PGTV. All four group plans required renormalization to achieve the same PGTV coverage and encompass at least 95% of the PTV.

**TABLE 2:** Treatment planning objectives and dose constraints for the planning target volumes (PTVs) and organs at risk (OARs)

**PTV/OAR**	**Parameter**	**Objective**
PGTV	D_98%_	>57 Gy
	D_2%_	<66 Gy
PCTV	D_98%_	>47.5 Gy
	D_2%_	<55 Gy
Both lungs	V_5Gy_	<65%
	V_20Gy_	<30%
	V_30Gy_	<20%
	D_mean_	<18 Gy
Spinal cord	D_max_	<45 Gy
Oesophagus	D_max_	<60 Gy
	D_mean_	<30 Gy
Heart	V_40Gy_	<30%
	D_mean_	<25 Gy

V*_x_* is the percentage volume of the OAR receiving at least *x* Gy of the radiation dose.

### 2.5 Dosimetric evaluation

Dose‐volume histograms were used to evaluate the dose distribution in the target and the dose volumes received by the OARs.

The parameters assessed for the PTVs included the conformity index (CI) and heterogeneity index (HI) of the target volume, dose received by 98% of the target volume (D_98%_), percentage of target volume covered by the prescribed dose (V_100%_), maximum dose (D_max_), and mean dose (D_mean_). As multiple‐dose gradients were used for PTVs, the HI was only calculated for the PGTV, and CI was calculated only for the PCTV. The CI and HI are defined as^18^, ^19^

(1) $$\mathrm{CI}\;\;=\;\frac{TV_{\mathrm{RI}}}{\mathrm{TV}}\;\times \frac{TV_{\mathrm{RI}}}{V_{\mathrm{RI}}}$$

(2) $$\mathrm{HI}\;\;=\;\frac{D_{2\%}-D_{98\%}}{D_{50\%}}$$

where TV is the volume of the PCTV (cm^3^), V_RI_ is the volume encompassed by the prescription isodose (cm^3^), TV_RI_ is the target volume covered by the prescription isodose (cm^3^), D_2%_, D_98%_, and D_50%_ represent the absolute doses covering the 2%, 98%, and 50% of the PGTV. A CI closer to 1 is indicative of better dose conformity of the PCTV, while a lower HI value indicates a more homogenous dose distribution within the PGTV.

For the serial‐type OARs, such as the spinal cord and esophagus, the maximum dose D_max_ for the individual plan was evaluated. For parallel‐type OARs, such as both lungs, esophagus, and heart, the mean dose D_mean_ and the percentage volume V_x_ covered by a particular dose were evaluated. The monitor units (Mus) of four plans were recorded to evaluate the beam utilization and execution efficiency.

### 2.6 Dose verification

Patient‐specific quality assurance was performed for each plan using an electronic portal imaging device. An absolute gamma index was conducted to compare the TPS‐calculated anisotropic analytical algorithm dose distribution with the measured 2D dose. The tolerance limit for gamma evaluation was 95% of points passing the criteria of 3% dose difference and 3 mm distance to agreement with a 10% threshold.

### 2.7 Statistical analysis

Pairwise comparisons of the dosimetric parameters in the four group plans were analyzed via the Wilcoxon test. All statistical analyses were performed using SPSS for Windows (version 26.0; IBM Corp, Armonk, NY, USA). Analysis items with *p* < 0.05 were considered statistically significant.

## 3 RESULTS

### 3.1 Comparison of dosimetric parameters

**FIGURE 2:**
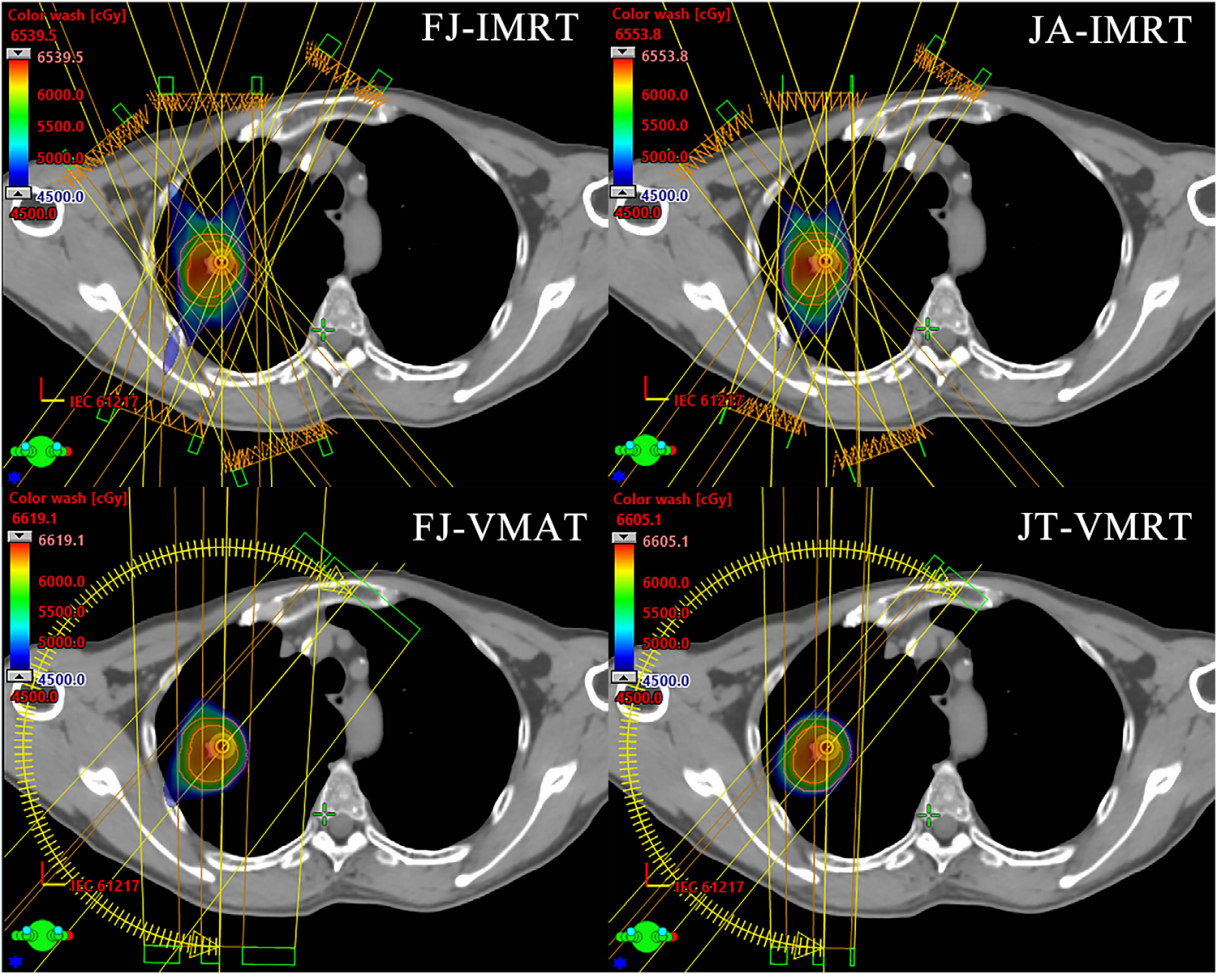
Isodose distributions for one patient with peripheral E lung cancer in four treatment plans: fixed jaw intensity‐modulated radiotherapy (FJ‐IMRT) = five beams IMRT plan with fixed jaw; adaptive jaw‐IMRT (JA‐IMRT) = five beams IMRT plan with adaptive jaw; fixed jaw volumetric‐modulated arc radiotherapy (FJ‐VMAT) = two reverse partial arcs VMAT plan with fixed jaw mode; and jaw tracking‐VMAT (JT‐VMAT) = two reverse partial arcs VMAT plan with jaw tracking mode

All four group plans met the requirement of a 95% prescribed dose coverage of the target volume. The characteristics of the dosimetric differences varied according to the classification of lung cancer. For all cases, the VMAT plans proved to have better CI than IMRT plans for all three types of lung cancer(*p* < 0.05; Figures 2 and 4).

**FIGURE 3:**
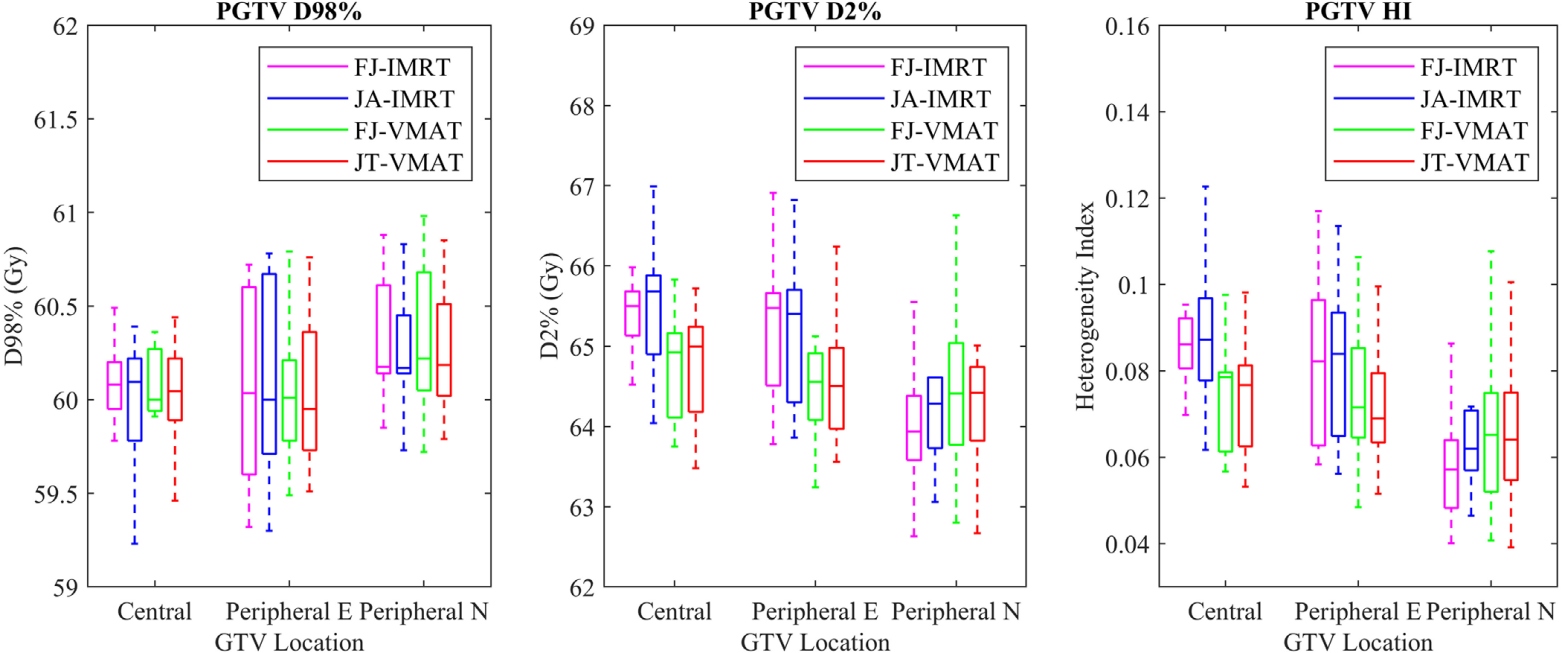
Dose parameter results of the planning gross tumor volume (PGTV) for lung cancer in four treatment plans

**FIGURE 4:**
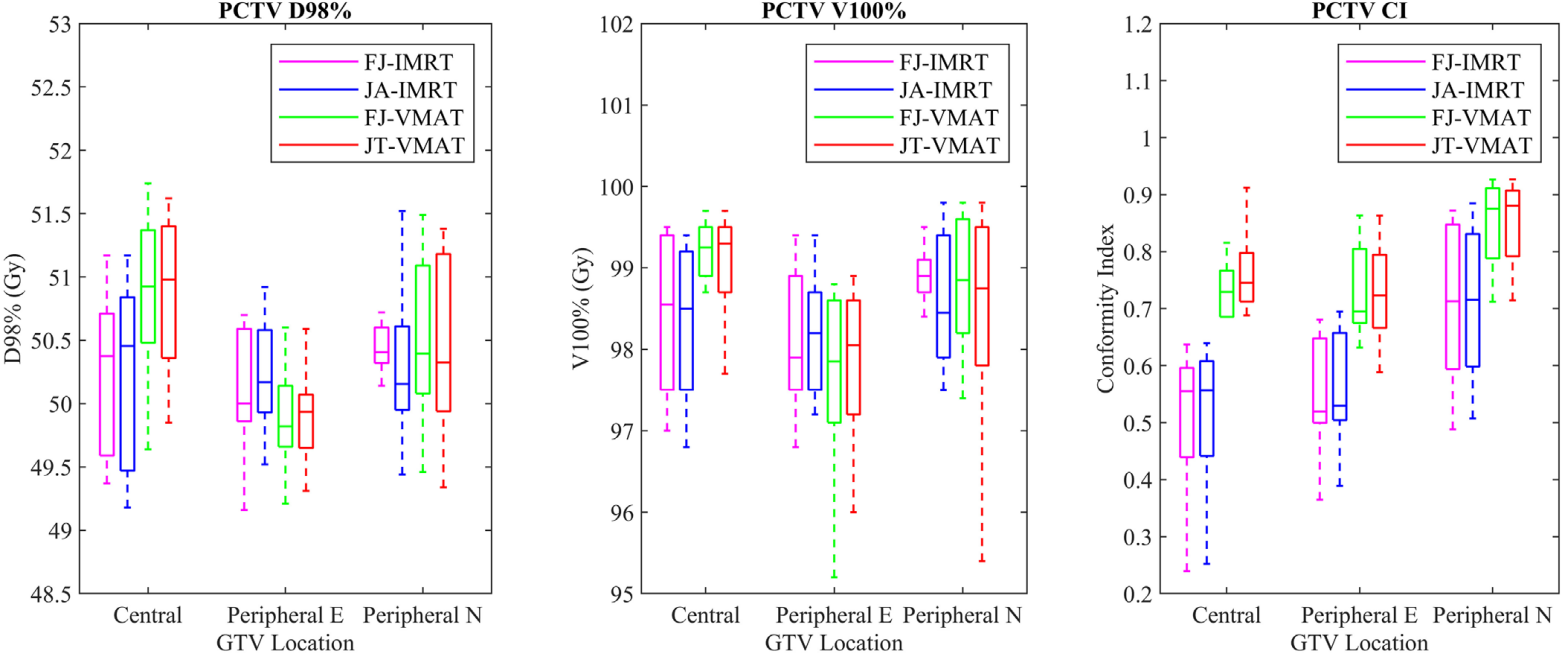
Dose parameter results of the planning clinical target volume (PCTV) for lung cancer in four treatment plans

**FIGURE 5:**
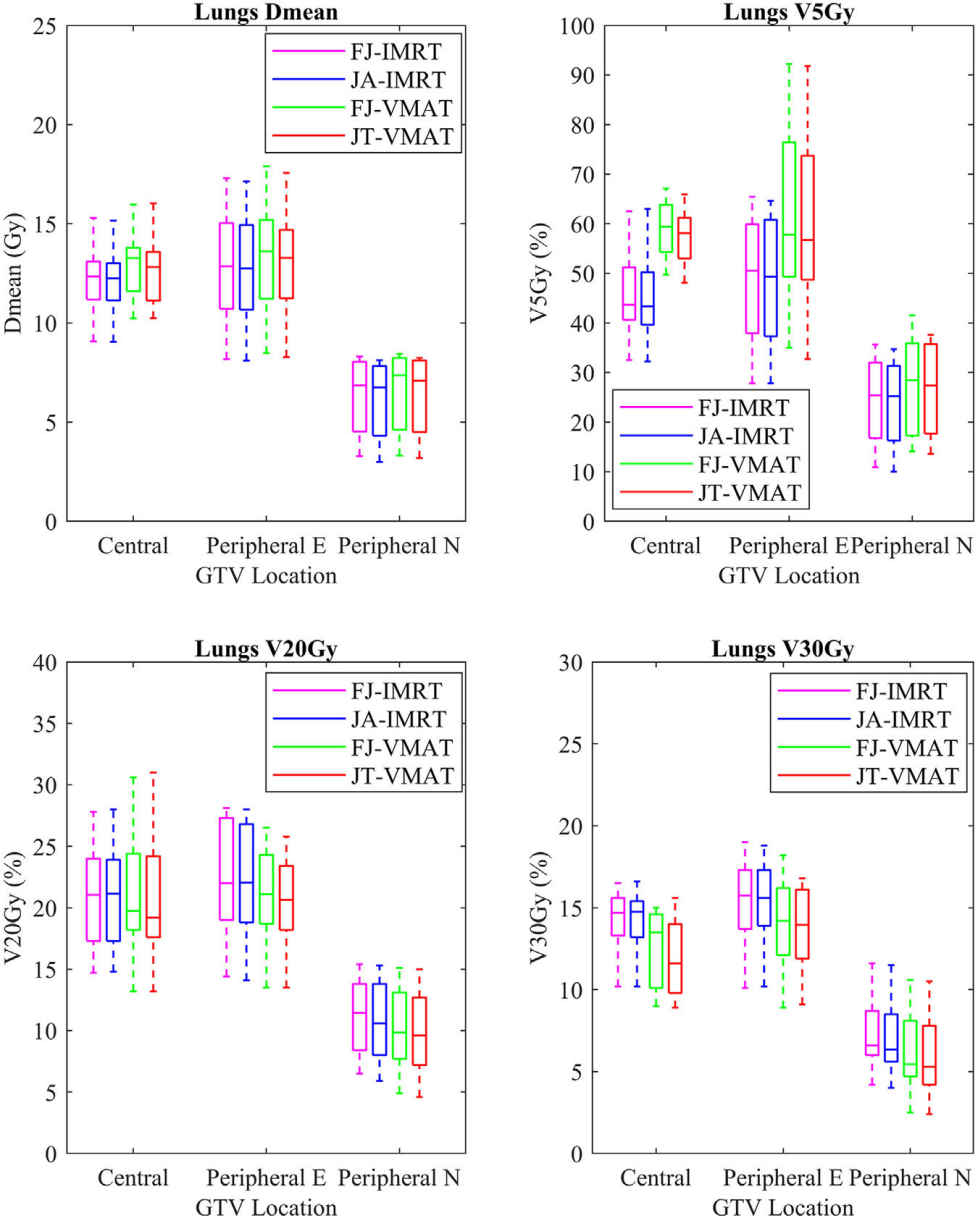
Dose parameter results of the total lung for lung cancer in four treatment plans

**FIGURE 6:**
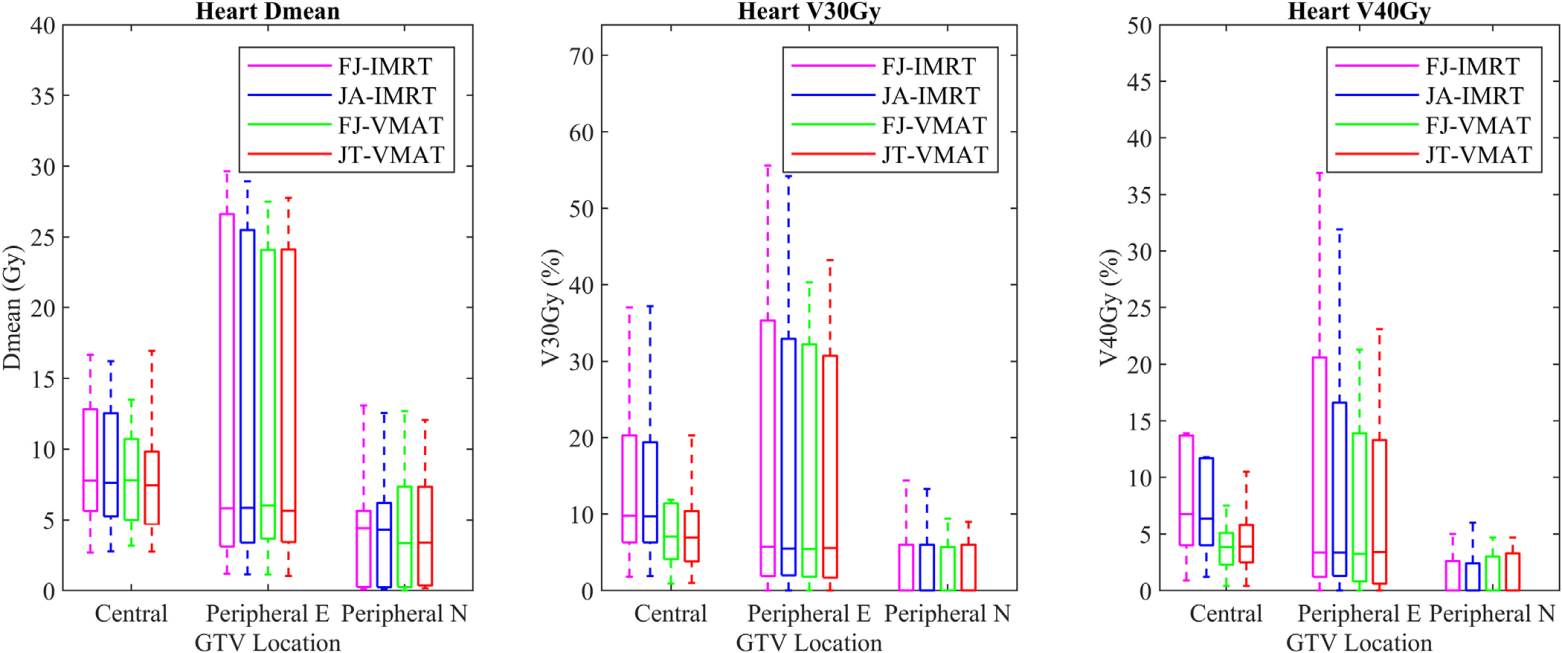
Dose parameter results of the heart in four treatment plans of each classification of lung cancer

**FIGURE 7:**
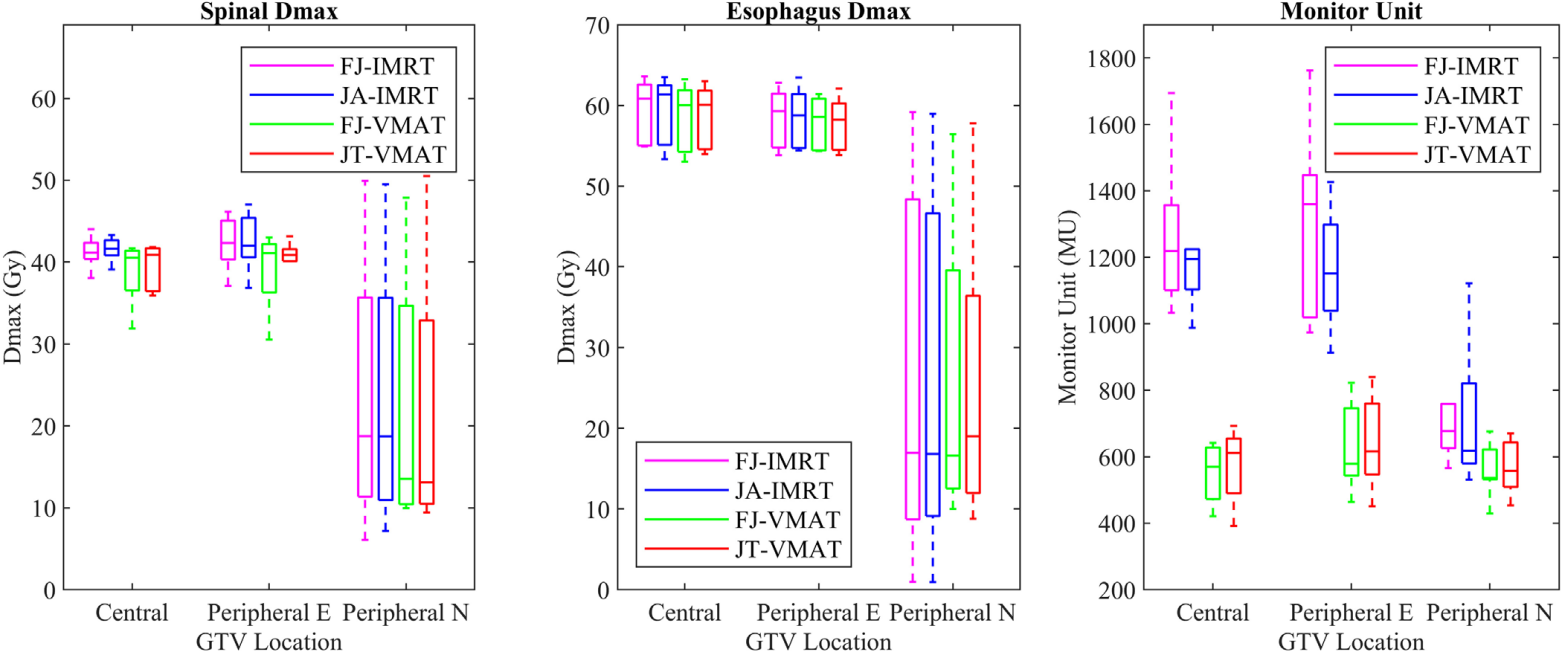
Dose parameter results for the spinal cord and esophagus and results of the machine monitor unit in four treatment plans of each classification of lung cancer

For the central lung cancer cases, the D_mean_ (Gy) and V_5Gy_ (%) of the total lung in the JA‐IMRT group were significantly lower than that in the FJ‐IMRT group (*p* < 0.05). The D_mean_ (Gy) and V_5Gy_ (%) of the total lung, and the D_mean_ (Gy) of the heart in the JT‐VMAT group were significantly lower than that in the FJ‐VMAT group (*p* < 0.05) (Figures 5 and 6; Table 3). Compared to the JA‐IMRT group, the VMAT groups had substantially lower irradiation dose to the heart and the V_30Gy_ (%) of the total lung (Figures 3 and  4; Table 3). The D_max_ of the spinal cord is 38.97 ± 3.28 (FJ‐VMAT) versus 41.48 ± 1.29 (JA‐IMRT) (*p* < 0.05). The JT‐VMAT group improved the HI (*p* < 0.05). However, the VMAT group increased the V_5Gy_ and D_mean_ of the total lung (*p* < 0.05) (Figure 7 and Table 3). There was no significant difference in the dose of the esophagus among the four groups.

**TABLE 3:** Comparison of dosimetric parameters among the four treatment groups for central lung cancer

**Parameter**	**FJ‐IMRT**	**JA‐IMRT**	**FJ‐VMAT**	**JT‐VMAT**	**P^I^**	**P^V^**	**P^T^**	**P^J^**
**PGTV**								
D_98%_ (Gy)	60.03 ± 0.29	60.00 ± 0.34	60.03 ± 0.29	60.03 ± 0.28	0.35	0.98	0.80	0.75
D_2%_ (Gy)	65.53 ± 0.72	65.52 ± 0.82	64.79 ± 0.70	64.76 ± 0.73	0.97	0.77	0.03	0.02
D_mean_ (Gy)	62.73 ± 0.35	62.74 ± 0.37	62.80 ± 0.45	62.79 ± 0.53	0.85	0.85	0.66	0.72
HI	0.09 ± 0.01	0.09 ± 0.02	0.08 ± 0.01	0.08 ± 0.01	0.84	0.83	0.05	0.04
**PCTV**								
D_98%_ (Gy)	50.25 ± 0.65	50.23 ± 0.70	50.87 ± 0.60	50.91 ± 0.62	0.79	0.54	0.02	0.01
V_100%_ (%)	98.40 ± 0.90	98.30 ± 0.90	99.10 ± 0.60	99.10 ± 0.70	0.22	0.92	0.02	0.02
CI	0.51 ± 0.12	0.52 ± 0.12	0.74 ± 0.11	0.72 ± 0.11	0.06	0.04	<0.01	<0.01
**Lungs**								
D_mean_ (Gy)	12.16 ± 1.85	12.06 ± 1.83	12.96 ± 1.74	12.63 ± 1.77	<0.01	<0.01	<0.01	0.03
V_5Gy_ (%)	46.20 ± 9.20	45.60 ± 9.20	59.00 ± 6.00	57.20 ± 5.90	0.02	<0.01	<0.01	<0.01
V_20Gy_ (%)	21.10 ± 4.10	21.10 ± 4.00	21.10 ± 4.90	20.90 ± 5.10	0.73	0.11	0.98	0.66
V_30Gy_ (%)	14.20 ± 2.10	14.20 ± 2.10	12.60 ± 2.30	12.00 ± 2.40	0.49	0.14	0.01	<0.01
**Heart**								
D_mean_ (Gy)	9.76 ± 6.49	9.67 ± 6.43	8.70 ± 4.88	8.14 ± 4.39	0.33	0.04	0.09	0.06
V_30Gy_ (%)	13.70 ± 11.20	13.30 ± 11.30	9.00 ± 7.80	7.80 ± 5.50	0.01	0.12	0.03	0.02
V_40Gy_ (%)	9.20 ± 8.80	8.70 ± 8.70	5.00 ± 5.10	4.20 ± 2.80	0.13	0.33	0.02	0.04
**Spinal cord**								
D_max_ (Gy)	41.24 ± 1.80	41.48 ± 1.29	38.97 ± 3.28	39.70 ± 2.55	0.46	0.53	0.04	0.05
**Esophagus**								
D_max_ (Gy)	54.48 ± 17.28	54.28 ± 17.48	53.92 ± 16.95	53.93 ± 17.27	0.43	0.96	0.23	0.33
D_mean_ (Gy)	24.41 ± 10.29	24.61 ± 10.47	24.78 ± 10.59	24.78 ± 10.65	0.10	0.96	0.74	0.71

*Notes*: P^I^: FJ‐IMRT vs. JA‐IMRT; P^V^: FJ‐VMAT vs. JT‐VMAT; P^T^: JA‐IMRT vs. FJ‐VMAT; P^J^: JA‐IMRT vs. JT‐VMAT.

Abbreviations: CI, conformity index; D_mean_, mean dose; D_max_, maximum dose; FJ‐IMRT, fixed jaw intensity‐modulated radiotherapy; FJ‐VMAT, fixed jaw volumetric‐modulated arc radiotherapy; HI, heterogeneity index; JA‐IMRT, jaw tracking intensity‐modulated radiotherapy; JT‐VMAT, jaw tracking volumetric‐modulated arc radiotherapy; PCTV, planning clinical target volume; PGTV, planning gross tumor volume.

**FIGURE 8:**
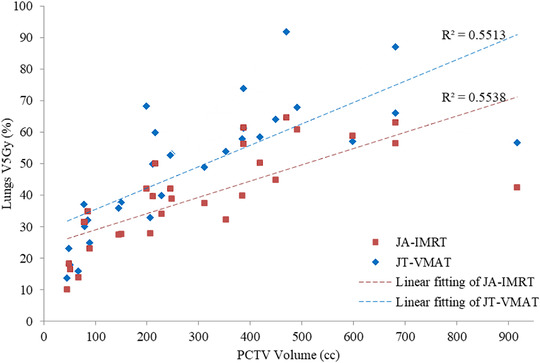
Relationship between the volume of the planning clinical target volume (PCTV) and V_5Gy_ (%) of the lungs in the adaptive jaw intensity‐modulated radiotherapy (JA‐IMRT) and jaw tracking volumetric‐modulated arc radiotherapy (JT‐VMAT) plans

For the peripheral E lung cancer cases, the JA‐IMRT group was superior to the FJ‐IMRT group in the CI (0.556 ± 0.096 vs. 0.546 ± 0.099, *p* < 0.05) (Figure 3 and Table 4). In comparison with the FJ‐VMAT group, the JT‐VMAT group significantly decreased the irradiation dose to the total lung (Figure 5 and Table 4). Compared to the JA‐IMRT group, the D_mean_ of the esophagus was lower in the VMAT group, the D_max_ of the spinal cord was lower in the FJ‐VMAT group, and the V_30Gy_ (%) of the total lung was lower in the JT‐VMAT group (*p* < 0.05) (Figure 7 and Table 4). However, the VMAT groups significantly increased the V_5Gy_ (%) of the total lung (Figure 5 and Table 4). As shown in Figure 8, the V_5Gy_ (%) of the total lung is positively correlated with the average volume of PCTV, and the V_5Gy_ (%) of the total lung in the VMAT group for some peripheral E lung cancer cases exceeded the dose constraint of 65% because of the huge PTV.

**TABLE 4:** Comparison of dosimetric parameters among the four treatment groups for peripheral E lung cancer

**Parameter**	**FJ‐IMRT**	**JA‐IMRT**	**FJ‐VMAT**	**JT‐VMAT**	**P^I^**	**P^V^**	**P^T^**	**P^J^**
**PGTV**								
D_98%_ (Gy)	60.08±0.49	60.11±0.5	60.01±0.40	60.05±0.44	0.20	0.36	0.27	0.40
D_2%_ (Gy)	65.31±0.94	65.31±0.95	64.63±0.89	64.55±0.79	0.91	0.37	0.06	0.03
D_mean_ (Gy)	62.84±0.49	62.83±0.53	62.59±0.72	62.59±0.68	0.85	0.99	0.36	0.33
HI	0.08±0.02	0.08±0.02	0.07±0.02	0.07±0.01	0.62	0.18	0.08	0.04
**PCTV**								
D_98%_ (Gy)	50.06±0.50	50.20±0.45	49.88±0.41	49.92±0.37	0.03	0.16	0.08	0.11
V_100%_ (%)	98.10±0.80	98.10±0.70	97.60±1.20	97.90±0.90	0.81	0.04	0.12	0.33
CI	0.55±0.10	0.56±0.10	0.73±0.08	0.73±0.08	0.02	0.87	<0.01	<0.01
**Lungs**								
D_mean_ (Gy)	12.82±2.99	12.76±2.96	13.21±2.93	12.91±2.82	0.20	<0.01	0.05	0.48
V_5Gy_ (%)	48.90±13.60	48.50±13.30	62.50±19.60	60.7±19.3	0.30	<0.01	<0.01	<0.01
V_20Gy_ (%)	21.80±5.10	21.70±5.10	20.70±4.60	20.30±4.30	0.82	<0.01	0.32	0.16
V_30Gy_ (%)	15.30±3.10	15.30±3.10	13.90±3.00	13.60±2.70	0.50	0.12	0.08	0.02
**Heart**								
D_mean_ (Gy)	11.96±11.76	11.75±11.33	11.40±10.42	11.34±10.75	0.21	0.89	0.51	0.08
V_30Gy_ (%)	16.90±21.30	16.50±20.50	13.80±15.90	14.20±16.90	0.18	0.74	0.21	0.17
V_40Gy_ (%)	10.90±14.0	9.70±12.10	6.90±7.90	7.00±8.10	0.11	0.95	0.14	0.11
**Spinal cord**								
D_max_ (Gy)	41.64±4.29	41.69±4.39	38.61±5.75	37.94±7.89	0.82	0.51	0.02	0.07
**Esophagus**								
D_max_ (Gy)	58.41±3.60	58.37±3.50	57.88±2.94	57.71±3.12	0.80	0.36	0.13	0.06
D_mean_ (Gy)	25.16±7.37	25.07±7.53	24.12±7.63	24.15±7.52	0.28	0.87	0.04	0.03

*Notes*: P^I^: FJ‐IMRT vs. JA‐IMRT; P^V^: FJ‐VMAT vs. JT‐VMAT; P^T^: JA‐IMRT vs. FJ‐VMAT; P^J^: JA‐IMRT vs. JT‐VMAT.

Abbreviations: CI, conformity index; D_mean_, mean dose; D_max_, maximum dose; FJ‐IMRT, fixed jaw intensity‐modulated radiotherapy; FJ‐VMAT, fixed jaw volumetric‐modulated arc radiotherapy; HI, heterogeneity index; JA‐IMRT, jaw tracking intensity‐modulated radiotherapy; JT‐VMAT, jaw tracking volumetric‐modulated arc radiotherapy; PCTV, planning clinical target volume; PGTV, planning gross tumor volume.

For the peripheral N lung cancer cases, the V_30Gy_ (%) and V_40Gy_ (%) of the heart were not listed since the value in most cases was almost zero. The JA‐IMRT group had better protection for total lung and esophagus than the FJ‐IMRT and JT‐VMAT groups had better protection for total lung than the FJ‐VMAT group (Figures 5 and 7; Table 5). Compared to the JA‐IMRT group, the V_30Gy_ (%) of the total lung was lower in the VMAT groups, but the D_mean_ and V_5Gy_ (%) of the total lung were higher (Figure 5 and Table 5). There was no significant difference in the dose of the heart, spinal cord, and esophagus among the four groups (P>0.05).

**TABLE 5:** Comparison of dosimetric parameters among the four treatment groups for peripheral N lung cancer

**Parameter**	**FJ‐IMRT**	**JA‐IMRT**	**FJ‐VMAT**	**JT‐VMAT**	**P^I^**	**P^V^**	**P^T^**	**P^J^**
**PGTV**								
D_98%_ (Gy)	60.30±0.32	60.23±0.34	60.32±0.41	60.25±0.33	0.19	0.21	0.10	0.62
D_2%_ (Gy)	63.94±0.85	64.31±0.76	64.49±1.04	64.39±0.91	0.03	0.16	0.27	0.58
D_mean_ (Gy)	62.32±0.50	62.44±0.45	62.70±0.58	62.60±0.46	0.14	0.09	0.02	0.07
HI	0.06±0.01	0.06±0.02	0.07±0.02	0.07±0.02	0.02	0.86	0.42	0.45
**PCTV**								
D_98%_ (Gy)	50.42±0.44	50.34±0.63	50.47±0.73	50.38±0.71	0.49	0.04	0.18	0.74
V_100%_ (%)	98.50±1.60	98.30±1.50	98.60±1.20	98.40±1.40	0.34	0.02	0.07	0.39
CI	0.71±0.14	0.71±0.14	0.85±0.08	0.86±0.07	0.92	0.23	<0.01	<0.01
**Lungs**								
D_mean_ (Gy)	6.34±1.78	6.13±1.83	6.51±1.95	6.35±1.86	<0.01	0.01	0.03	0.07
V_5Gy_ (%)	24.10±8.50	23.30±8.40	27.60±10.10	26.70±9.00	<0.01	0.146	0.02	0.03
V_20Gy_ (%)	11.10±3.10	10.80±3.20	10.30±3.40	10.00±3.50	0.08	0.04	0.24	0.07
V_30Gy_ (%)	7.20±2.30	6.90±2.30	5.90±2.50	5.70±2.50	0.03	<0.01	<0.01	<0.01
**Heart**								
D_mean_ (Gy)	4.28±4.31	4.25±4.18	4.34±4.39	4.41±4.32	0.76	0.68	0.13	0.17
**Spinal cord**								
D_max_ (Gy)	22.95±15.30	23.00±15.05	20.99±13.62	20.38±14.28	0.77	0.41	0.44	0.33
**Esophagus**								
D_max_ (Gy)	23.52±20.94	22.77±20.74	24.1±16.37	24.41±16.48	0.25	0.69	0.49	0.40
D_mean_ (Gy)	4.63±5.40	4.49±5.43	5.41±3.90	5.28±3.78	0.03	0.45	0.16	0.22

*Notes*: P^I^: FJ‐IMRT vs. JA‐IMRT; P^V^: FJ‐VMAT vs. JT‐VMAT; P^T^: JA‐IMRT vs. FJ‐VMAT; P^J^: JA‐IMRT vs. JT‐VMAT.

Abbreviations: CI, conformity index; D_mean_, mean dose; D_max_, maximum dose; FJ‐IMRT, fixed jaw intensity‐modulated radiotherapy; FJ‐VMAT, fixed jaw volumetric‐modulated arc radiotherapy; HI, heterogeneity index; JA‐IMRT, jaw tracking intensity‐modulated radiotherapy; JT‐VMAT, jaw tracking volumetric‐modulated arc radiotherapy; PCTV, planning clinical target volume; PGTV, planning gross tumor volume.

### 3.2 Comparison of MUs

The MUs for each plan were based on the calculations of the treatment planning system. The VMAT plans had considerably fewer MUs than the IMRT plans for both the central and peripheral cases (*p* < 0.05). In comparison with the FJ‐IMRT technique, the JA‐IMRT technique had more MUs, and in comparison with the FJ‐VMAT technique, the JT‐VMAT technique had more MUs (*p* < 0.05) (Figure 7 and Table 6).

**TABLE 6:** Mean monitor units (MUs) of each technique

**Classification**	**FJ‐IMRT**	**JA‐IMRT**	**FJ‐VMAT**	**JT‐VMAT**
Central	1207±173	1259±205	556±81	585±100
Peripheral E	1226±287	1307±261	618±131	642±127
Peripheral N	722±222	772±238	563±74	566±72

Abbreviations: FJ‐IMRT, fixed jaw intensity‐modulated radiotherapy; FJ‐VMAT, fixed jaw volumetric‐modulated arc radiotherapy; JA‐IMRT, jaw tracking intensity‐modulated radiotherapy; JT‐VMAT, jaw tracking volumetric‐modulated arc radiotherapy.

### 3.3 Quality assurance pass rates

Table 7 showed that the gamma passing rate in all plans is above 95%, meeting the standards of our institution requirements. Compared with the FJ‐VMAT group, the JT‐VMAT group has a higher passing rate of dose verification.

**TABLE 7:** Gamma passing rates (%) among the four treatment groups under criteria of 3% dose difference (DD) and 3 mm distance to agreement (DTA) with 10% threshold

**Classification**	**FJ‐IMRT**	**JA‐IMRT**	**FJ‐VMAT**	**JT‐VMAT**	**P^I^**	**P^V^**	**P^T^**	**P^J^**
Central	99.5±0.4	99.4±0.8	96.9±2.1	99.6±0.4	0.72	<0.01	0.01	0.70
Peripheral E	99.6±0.3	99.9±0.1	98.0±1.9	99.5±0.5	<0.01	0.01	0.01	0.02
Peripheral N	100.0±0.0	100.0±0.0	97.7±1.1	99.5±0.4	1.0	<0.01	<0.01	<0.01

*Notes*: P^I^: FJ‐IMRT vs. JA‐IMRT; P^V^: FJ‐VMAT vs. JT‐VMAT; P^T^: JA‐IMRT vs. FJ‐VMAT; P^J^: JA‐IMRT vs. JT‐VMAT.

Abbreviations: FJ‐IMRT, fixed jaw intensity‐modulated radiotherapy; FJ‐VMAT, fixed jaw volumetric‐modulated arc radiotherapy; JA‐IMRT, jaw tracking intensity‐modulated radiotherapy; JT‐VMAT, jaw tracking volumetric‐modulated arc radiotherapy.

## 4 DISCUSSION

This study verified the superior CI and significantly reduced MUs of the VMAT technique in comparison with that in the IMRT technique, as proposed in several articles.^20^, ^21^, ^22^ Compared to the IMRT groups, the VMAT groups had substantially lower irradiation doses to most OARs, especially total lung (V_20Gy_ and V_30Gy_) and heart. However, VMAT increased the V_5Gy_ of the total lung. As shown in Figure 8, the V_5Gy_ (%) of the total lung is positively correlated with the average volume of PCTV. When the volume of PCTV is the same, V_5Gy_ (%) of the total lung in the JT‐VMAT group is higher than that in the JA‐IMRT group, and the degree increased with the increase of the volume of PCTV. When the PTV volume exceeds about 400 cm^3^, the V_5Gy_ (%) of the lungs for the VMAT group probably exceed the clinical dose constraint (<65%).

In our study, we discovered that the VMAT technique could significantly reduce V_30Gy_ and V_40Gy_ of the heart for central lung cancer, and thus, patients with poor cardiac function may benefit from VMAT.^23^ Because of the huge target volume, the V_5Gy_ of the total lung of VMAT plans in some cases from the peripheral E lung cancer group exceeded the clinical dose constraint and the parameter was close to 65% in some cases from central lung cancer. The correlation between radiation pneumonitis and dosimetric constraints has been validated, and a cutoff of 65% for V5 (%) of the total lung were sensitive to radiation pneumonitis.^24^, ^25^ In actual clinical practice, to prevent the occurrence of radiation pneumonia when the planned dose to the lung exceeds the clinical dose constraint, a method of lowering the dose coverage of the target area is typically applied to maintain the dose irradiated to the lung below the dose limit, which may reduce the rate of tumor control. Therefore, for peripheral E lung cancer, the VMAT techniques should be used carefully, particularly for patients with large targets and poor pulmonary function.

In comparison with FJ‐IMRT, JA‐IMRT significantly reduced the dose irradiated to total lung for the central lung cancer, improved the CI for the peripheral E lung cancer, and reduced radiation dose to the total lung and esophagus for the peripheral N lung cancer. Mani et al.^26^ also demonstrated that the dose reductions were observed for OARs in JA‐IMRT for head and neck cancer. This may be because the area of OARs irradiated and the volume outside the PTV irradiated are reduced by manual optimization of collimator angle and jaw positions. Moreover, all IMRT fields with collimator rotation of 0° would mean that transmission due to the tongue‐and‐groove effect would be summed up unfavorably for all the IMRT fields. In comparison with the FJ‐VMAT technique, the JT‐VMAT technique provided better protection for the total lung, thereby reducing the incidence of radiation pneumonia.^27–13^ Moreover, JT‐VMAT significantly reduced the D_mean_ of the heart for central lung cancer. Pokhrel et al.^28^ discovered that there was no significant difference in the dose of the heart between FJ‐VMAT and JT‐VMAT, which was different from our results. This may be attributable to the fact that they did not categorize the cancer cases by location. It is evident that the JT technique can reduce the x‐ray transmission from the MLC and the leakage between the MLCs. The extent of the reduction may be related to the location and volume of the target being irradiated. In addition, our results confirmed that the JA‐IMRT/ JT‐VMAT groups could slightly increase treatment plan MUs which were consistent with the previous study.^29^

There are several limitations in the present study that we should pay attention to. First, the number of patients for the current research were small, since only 10 cases from each category of lung cancer were involved, and all of the baseline characteristics might have a possible adverse influence. In addition, the treatment plan quality of IMRT is typically strongly dependent on the number of fields and angle of beams. In our study, in order to obtain an ideal dose distribution, we used five fields for IMRT plans and designed the beam angle based on tumor locations. This process was merely based on the past experience of our cancer center, lack of strict evidence, and it was difficult to say that the best IMRT plan was obtained. Furthermore, on the selection of radiotherapy techniques, this investigation focused on dose‐volume parameters and data from literature reports, lack of actual dose verification, and follow‐up results.

## 5 CONCLUSION

In summary, when choosing a therapeutic technique for lung cancer radiotherapy, dosimetry and clinical complications should be considered comprehensively. For central lung cancer with normal pulmonary function, we advise VMAT techniques, particularly the JT‐VMAT technique, for better CI, fewer MUs, and lower dose irradiated to the heart. Conversely, for central lung cancer with poor pulmonary function, JA‐IMRT was recommended to avoid radiation pneumonia. For peripheral E lung cancer, we advocate the JA‐IMRT technique to keep V_5Gy_ of the total lung under the constraint. For peripheral N lung cancer, the VMAT techniques are strongly recommended to reduce MUs and obtain a better CI, and the JT‐VMAT technique should be the first choice as long as the facility conditions permit.

## CONFLICT OF INTEREST

The authors declare that they have no conflict of interest.

## FUNDING INFORMATION

National Natural Science Foundation of China, Grant/Award Number: 12075329, 12005316; Guangdong Esophageal Cancer Institute Science, Grant/Award Number: Q201907, Q201908; Cancer Precision Radiotherapy Spark Program of China International Medical Foundation, Grant/Award Number: 2019‐N‐11‐20.

## AUTHOR CONTRIBUTIONS

Li Chen designed and supervised the study. Qinghe Peng, Jun Zhang, and Zhenghuan Li designed the treatment plans. Qinghe Peng and Qingyuan Zhang collected and analyzed the data. Jun Zhang and Qiwen Li provided technical assistance for the study. Yinglin Peng and Qinghe Peng wrote the manuscript. All co‐authors have reviewed and approved the final manuscript.

## ETHICAL APPROVAL AND CONSENT TO PARTICIPATE

Not applicable

## Data Availability

The datasets are backed up on the Research Data Deposit (RDD Number: RDDA2021001938, https://www.researchdata.org.cn) and are available upon reasonable request.
